# Association between maternal iron supplementation and newborn birth weight: a quantile regression analysis

**DOI:** 10.1186/s13052-021-01084-7

**Published:** 2021-06-05

**Authors:** Guoshuai Shi, Zhuo Zhang, Lu Ma, Binyan Zhang, Shaonong Dang, Hong Yan

**Affiliations:** 1 Department of Epidemiology and Biostatistics, School of Public health, Xi’an Jiaotong University Health Science Center, Xi’an, 710061 Shaanxi China; 2 Institute of Chinese Medical Sciences & State Key Laboratory of Quality Research in Chinese Medicine, University of Macau, Macau, China; 3 Xi’an Shiyou University, Xi’an, 710065 Shaanxi China; 4 Nutrition and Food Safety Engineering Research Center of Shaanxi Province, Xi’an, 710061 China

**Keywords:** Neonatal birth weight, Iron supplementation, Quantile regression, Northwest China

## Abstract

**Objective:**

Our study aimed to explore the association between maternal iron supplementation and newborn birth weight (BW) in Shaanxi Province using quantile regression (QR).

**Method:**

The data used in this study were derived from a large cross-sectional survey of a population in Shaanxi Province, Northwest China. A total of 28,209 women and their infants were selected using a stratified multistage random sampling method. The effect of iron supplementation on the newborn BW was assessed by a multiple linear regression model and QR.

**Results:**

A total of 5.15% of the women took iron supplements during pregnancy. Multiple linear regression showed that the iron supplementation during pregnancy had positive effects on the BW, with an average increase of 43.07 g (β = 43.07, t = 3.55, and *p* < 0.001). The QR showed that the iron supplementation during pregnancy was associated with an increased newborn BW from very low to higher percentiles (quantiles: 0 ~ 0.40), with the β ranging from 136.51 to 43.86. As the percentiles of the BW increased, the neonatal BW gain gradually declined in the iron supplementation group compared with the group that did not receive iron supplementation (quantiles: 0 ~ 0.40, with the β ranging from 136.51 to 43.86). Iron supplementation was more effective among women who suffered from anemia during pregnancy (β = 45.84, t = 2.05, and *p* = 0.04; quantiles: 0 ~ 0.15, 0.30, 0.80, with β ranging from 150.00 to 39.29) than it was in any other group (β = 38.18, t = 2.62, and *p* = 0.009; quantiles: 0 ~ 0.15, with β ranging from 133.33 to 28.32).

**Conclusions:**

Iron supplementation during pregnancy is associated with an increased newborn BW, and the effect was more obvious in the newborns with the lower BW and newborns whose mothers suffered from anemia during pregnancy.

**Supplementary Information:**

The online version contains supplementary material available at 10.1186/s13052-021-01084-7.

## Introduction

Birth weight (BW) is considered a significant indicator for assessing fetal intrauterine growth, the nutritional condition and the health status of newborns, as well as predicting growth, developmental status, and health problems in adulthood [1–3]. In newborns with a normal weight, those with a low birth weight and macrosomia are related to higher morbidity and mortality [4–6], and low birth weight can result in poor mental development, risk of infection, and risk of developing cardiovascular disease later in life [7–9].

During pregnancy, due to fetal growth and maternal physiological changes, the demand for micronutrients increases [10], but obtaining adequate micronutrients from a daily diet is difficult [11]; further, micronutrient deficiency in mothers can lead to serious adverse consequences, such as low birth weight [12]. To meet the increased nutritional demands during pregnancy, routine intake of certain micronutrients that are critical to the health of mothers and newborns during pregnancy is strongly recommended, which also has favorable delivery outcomes [13–16]. The WHO recommends that pregnant women should take daily oral iron (30 mg to 60 mg) and folic acid (0.4 mg) supplements to avoid maternal anemia, preterm delivery, and low birth weight [17]. Iron is an essential component of hemoglobin and myoglobin, accounting for approximately 60% of the body’s iron. The iron requirement triples during pregnancy due to the growth of the fetoplacental units and the increase in the number of maternal red blood cells [18]. Several studies explored the relationship between iron supplementation and the BW [19, 20]. Most countries recommend routine iron supplementation during pregnancy. However, routine prenatal iron supplementation is not recommended in China due to the possibility of iron supplementation during pregnancy resulting in gastrointestinal side effects and adverse birth outcomes [21].

Although extensive studies suggest a valuable relationship between iron supplementation and BW [14, 18, 22], the relationship is not fully elucidated. To our knowledge, there has not been research using quantile regression (QR) to explore the relationship between iron supplementation and BW. Whether the logistic regression with BW as a categorical variable or the linear regression with BW as a continuous variable, the reported models only described the conditional mean value of the results but failed to describe the scale and classification of the distribution, which also led to a lack of information [23]. However, the QR approach in this study offers inferences on multiple quantiles of the BW distribution that would be more informative than the inference on the mean BW alone [24]. This method has statistical efficiency and robustness regarding outliers without any requirement for random error distribution [25, 26]. In addition, there has not been a large population study to analyze the association of iron supplementation and newborn BW. This study aims to overcome these limitations. Therefore, the purpose of this study was to use population-based large-scale sample survey data to describe the effect of iron supplementation on the overall BW distribution through the QR, thus providing valuable information to inform health care programs and policies.

## Methods

### Study design and participants

The data used in this study were derived from a large cross-sectional survey of a population in Shaanxi Province, Northwest China, between August and November 2013. The survey was designed to investigate the risk factors affecting adverse birth outcomes. Considering the different proportions and fertility rates in the urban and rural residents, this study adopted a stratified multistage random sampling design to select women of childbearing age (15 ~ 49) who gave birth, lived in the research area and had definitive pregnancy outcomes in 2010 ~ 2013. Women who experienced serious illnesses, such as cardiovascular disease or cancer, during the investigation, were excluded. The sampling method was comprehensively presented in the previous literature [27]. Briefly, 10 districts and 20 counties were randomly selected from the urban and rural strata, respectively. Then, we randomly sampled three streets from each sampled district and six communities from each street in the urban area, and six townships from each sampled county, and six villages from each township in the rural area. Finally, we randomly selected 60 eligible newborns and their mothers in each sampled community and 30 in each sampled village.

### Data collection

After acquiring written informed consent, well-trained investigators conducted in-person interviews to collect the information about women using a structured questionnaire, including their place of residence, date of birth, educational level, family economic status, pregnancy history, prenatal care, lifestyle, and health status, and information was also collected about children, including gender and fetal number. BWs were measured to the nearest 10 g, and birth dates were obtained by reviewing the birth certificates. Before the investigation, the investigators received unified training, passed the examination, and mastered the inquiry skills and questionnaire filling abilities. To guarantee the quality of the survey, we reviewed the questionnaires at three levels: on-site review was performed by investigators; scrutiny after the day of investigation was performed by investigators who exchanged and checked the questionnaires; and the supervisors checked each questionnaire to see if there were any missing values or logical errors and returned to reinvestigation if any errors were found. At the end of each district (county) survey, 5% of the respondents were randomly selected for repeated surveys to ensure the authenticity and credibility of the data.

There were 30,027 women enrolled in the survey. After excluding non-live births and multiple gestations (*n* = 1116), and unknown BW and iron supplementation status (*n* = 702), 28,209 single live newborns and their mothers were finally included as the sample population.

### Study variables

Newborn BW was used as the outcome variable in this analysis. The BW, which was measured within 1 h after birth, was collected through browsing the birth certificates and operationalized as a continuous variable. For the comparison between groups, the BW was separated into three categories: normal BW (2500 g ~ 4000 g), low birth weight (< 2500 g), and macrosomia (> 4000 g). Iron supplementation was considered as the exposure variable. Women were divided into two groups: the iron supplementation group (iron supplementation, such as ferrous sulfate tablets, iron dextran tablets, from 3 months before pregnancy to the end of pregnancy) and the non-users (no iron supplementation). One to three months before pregnancy was defined as before pregnancy, and 1 ~ 3, 4 ~ 6, and 7 ~ 10 months of pregnancy were defined as the first trimester, the second trimester, and the third trimester, respectively.

### Covariates

Several factors were defined as covariates in this study. The covariates were classified as follows: maternal age (< 25, 25 ~ 29 or > 30 years); residence (urban area or rural area); family economic status (low, middle or high); mother’s education (primary school or less, junior high school or senior high school or above); passive smoking (yes or no); folate supplementation (yes or no); pregnancy-induced hypertension (PIH; yes or no); anemia (yes or no); parity (1 or ≥ 2); medication use (yes or no); number of antenatal care (ANC) visits (< 5 or ≥ 5); preterm status (yes or no); and neonatal sex (boy or girl). Maternal age was calculated from the birth date of the woman to the birth date of the child. Family economic status was calculated as follows: family adjusted adult number = family adults number + 0.5 × family children number, per capita annual household income = annual household income/family adjusted adult number. Family economic status was classified as low, middle, and high according to the upper quartile and lower quartile of per capita annual household income. Passive smoking was referred to as exposure to tobacco smoke from others for at least 15 min/d [28]. Anemia and PIH were self-reported by participants, and evaluation of anemia and PIH was performed according to whether women were diagnosed by their physicians during pregnancy. Preterm birth was defined as birth at < 37 weeks of gestation. Medication mainly included cold medicine, antibiotics, antidepressants, salicylic acid drugs, and antitussives.

### Statistical analysis

All data were double-entered into a database established using EpiData3.1 (EpiData Association, Denmark). The characteristics of the subjects were expressed as the mean ± standard deviation for the continuous variables and counts (proportions) for the categorical variables. In the univariate analysis, a *t*-test or *χ*^*2*^ test was employed for comparisons between groups.

Ordinary least squares (OLS) and the QR were employed to assess the association of iron supplementation with the mean and the different quantiles (0.05, 0.10, 0.15, 0.20, 0.25, 0.30, 0.35, 0.40, 0.45, 0.50, 0.55, 0.60, 0.65, 0.70, 0.75, 0.80, 0.85, 0.90, and 0.95) of the BW, adjusted for the covariates. The graphs were plotted to visualize the changes in the effect of iron supplementation on the BW at different percentiles. The 95% CI of the regression coefficient was expressed by the shading around the curve. Considering the low intake rate of iron before pregnancy, we combined the group of women supplemented with iron before pregnancy and in the first trimester into a single group when analyzing the association of iron supplementation with the BW at different periods. Moreover, as a sensitivity analysis, the stratified analysis was conducted based on the anemia status. Stata 15.1 software (StataCorp) was used for all analyses.

## Results

### Background information of the participants

The baseline characteristics of the 28,209 women surveyed according to iron supplementation are displayed in Table 1. Among the women surveyed, the reproductive age was 26.56 ± 4.75 years. About four-fifths live in rural areas (79.24%). The mother’s education level was mainly concentrated in junior high school and above (88.10%). The per capita annual household income of the participants with low, middle, and high economic status was 4946, 9029, and 13,600 yuan, respectively. A total of 67.65% had the supplementation of folic acid, and 16.34% had taken drugs. During pregnancy, 1.56, 16.35, and 24.70% suffered from PIH, anemia, and second-hand smoke exposure, respectively. A total of 67.91% had 5 or more ANC visits. The proportions of the different parities were 59.26% (1) and 40.74% (≥2). Of the infants, 5.98% were premature, and 54.83% were boys.

**Table 1:** Characteristics of the study participants^a^

Baseline characteristics	Non-users	Iron supplementation	t/ χ^2^	*P* values
Maternal age, year^b^	26.53 (4.77)	27.03 (4.37)	−3.91	<0.001
Residence			143.40	<0.001
Urban area	5375 (20.09)	482 (33.17)		
Rural area	21,381 (79.91)	971 (66.83)		
Economic status			70.42	<0.001
Low	5123(19.15)	223(15.35)		
Middle	15,293(57.16)	747(51.41)		
High	6340(23.70)	483(33.24)		
Mother’s education			328.94	<0.001
Primary school or less	3299(12.33)	57(3.92)		
Junior high school	13,481(50.38)	521(35.86)		
Senior high school or above	9976(37.29)	875(60.22)		
Passive smoking			36.60	<0.001
No	20,051(74.94)	1191(81.97)		
Yea	6705(25.06)	262(18.03)		
Folate supplementation			71.66	<0.001
No	8802(32.90)	323 (22.23)		
Yea	17,954 (67.10)	1130 (77.77)		
PIH			0.02	0.894
No	26,339 (98.44)	1431 (98.49)		
Yea	417 (1.56)	22 (1.51)		
Anemia			397.35	<0.001
No	23,021 (86.04)	972 (66.90)		
Yea	3735 (13.96)	878 (33.10)		
Parity			65.73	<0.001
1	15,709 (58.71)	1009 (69.44)		
≥ 2	11,047 (41.29)	444 (30.56)		
Medication use			31.15	<0.001
No	22,461 (83.95)	1139 (78.39)		
Yes	4295 (16.05)	314 (21.61)		
Number of ANC visits			184.28	<0.001
<5	8821 (32.97)	231 (15.90)		
≥ 5	17,935 (67.03)	1222 (84.10)		
Preterm status			7.50	0.006
No	25,180 (94.11)	1342 (92.36)		
Yes	1576 (5.89)	111 (7.64)		
Neonatal sex			0.09	0.761
Boy	14,675 (54.85)	791 (54.44)		
Girl	12,081 (45.15)	662 (45.56)		

*ANC* Antenatal care, *PIH* Pregnancy-induced hypertension; ^a^Reported as n (%); ^b^Values were presented as mean and SD for the continuous variable

### Status of the neonatal BW and iron supplementation

The BW status of the newborns in the two groups is shown in Table 2. Significant differences existed in the distribution of the BW in the iron supplementation group and the no-users group. The average BW of 28,209 children was 3267.21 ± 459.97 g. The prevalence of low birth weight and macrosomia was 3.41 and 6.85%, respectively. The neonatal average BWs of the women with iron supplementation and without iron supplementation were 3326.02 ± 442.60 g and 3264.01 ± 460.68 g, respectively. Newborns in the iron supplementation group had a higher average BW than those in the no-users group (*p* < 0.001). A total of 1453 (5.15%) women of childbearing age had the supplementation of iron during pregnancy. The rates of iron supplementation at different stages were 0.26% (74/28209, before pregnancy), 1.55% (437/28209, the first trimester), 2.64% (745/28209, the second trimester), and 2.34% (661/28209, the third trimester).

**Table 2:** Newborn birth weight of iron supplementation and no-users

	N	Normal	Low birth weight	Macrosomia	BW
No-users	26,756	24,026 (89.80)	925 (3.46)	1805 (6.75)	3264.01 ± 460.68
Iron supplementation	1453	1287 (88.58)	38 (2.62)	128 (8.81)	3326.02 ± 442.60
Total	28,209	22,426(89.73)	963 (3.41)	1933 (6.85)	3267.21 ± 459.97
χ^2^/ t	11.65				−5.01
*P* value	0.003				< 0.001

*BW* Birth weight

### Association between iron supplementation and the neonatal BW

**Fig. 1:**
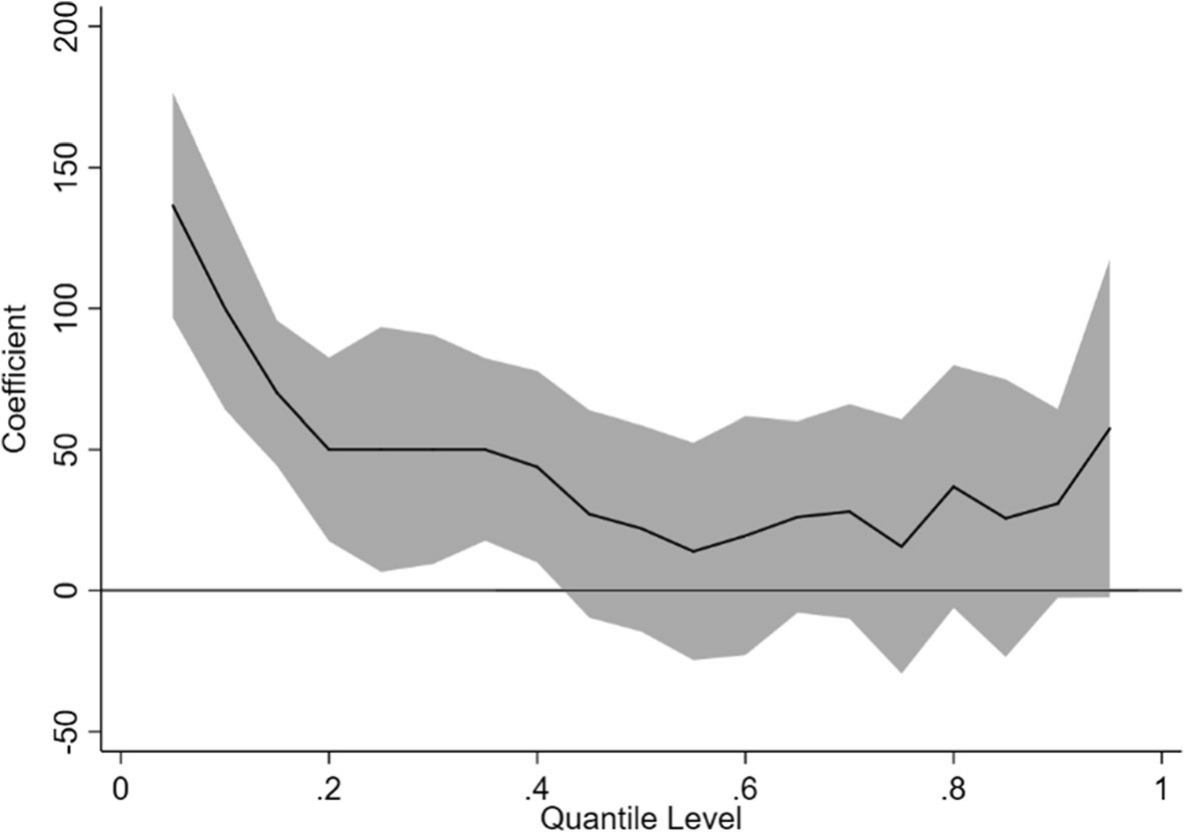
Graphical illustration of the effect of iron supplementation on birth weight in different percentiles

Table 3 and Fig. 1 show the comparison of the association between iron supplementation (independent variable) and BW (dependent variable) using the QR and OLS methods with the confounding factors controlled. The QR coefficients indicated that the effects of the QR on neonatal BW were different in the different distribution parts. Multiple linear regression indicated that the iron supplementation during pregnancy had positive effects on the BW, with an average increase of 43.07 g (β = 43.07, t = 3.55, and *p* < 0.001). However, the multiple linear regression analysis could not fully explain the relationship between iron supplementation and newborn BW. The QR indicated that the iron supplementation during pregnancy was associated with an increased newborn BW from very low to higher percentiles (quantiles: 0 ~ 0.40), with β ranging from 136.51 to 43.86, and the difference was significant. As the percentiles of the BW increased, the neonatal BW gain gradually declined in the iron supplementation group compared with the newborns without iron supplementation.

**Table 3:** QR and OLS results for accessing the association between iron supplementation and birth weight in different percentiles

Quantile	β	95% CI	t	*P* values
0.05	136.51	96.58 ~ 176.44	6.70	< 0.001
0.10	100.00	64.09 ~ 135.91	5.46	< 0.001
0.15	70.00	44.27 ~ 95.73	5.33	< 0.001
0.20	50.00	17.48 ~ 82.52	3.01	0.003
0.25	50.00	6.59 ~ 93.41	2.26	0.024
0.30	50.00	9.40 ~ 90.60	2.41	0.016
0.35	50.00	17.69 ~ 82.31	3.03	0.002
0.40	43.86	9.93 ~ 77.80	2.53	0.011
0.45	27.10	−9.67 ~ 63.86	1.44	0.149
0.50	22.01	−14.63 ~ 58.65	1.18	0.239
0.55	13.85	−24.74 ~ 52.44	0.70	0.482
0.60	19.41	−22.95 ~ 61.77	0.90	0.369
0.65	26.05	−7.91 ~ 60.01	1.50	0.133
0.70	28.00	−10.02 ~ 66.02	1.44	0.149
0.75	15.53	−29.56 ~ 60.63	0.68	0.500
0.80	36.84	−6.18 ~ 79.86	1.68	0.093
0.85	25.60	−23.62 ~ 74.82	1.02	0.308
0.90	30.83	−2.58 ~ 64.25	1.81	0.071
0.95	57.47	−2.40 ~ 117.34	1.88	0.060
OLS	43.07	19.31 ~ 66.83	3.55	< 0.001

*QR* Quantile regress, *OLS* Ordinary least squares, QR and multiple linear regression analysis adjusted for maternal characteristics (including maternal age, residence, economic status, mother’s education, passive smoking, folate supplementation, pregnancy-induced hypertension, anemia, parity, medication use, number of ANC visit) and neonatal characteristics (including preterm status and neonatal sex)

**Fig. 2:**
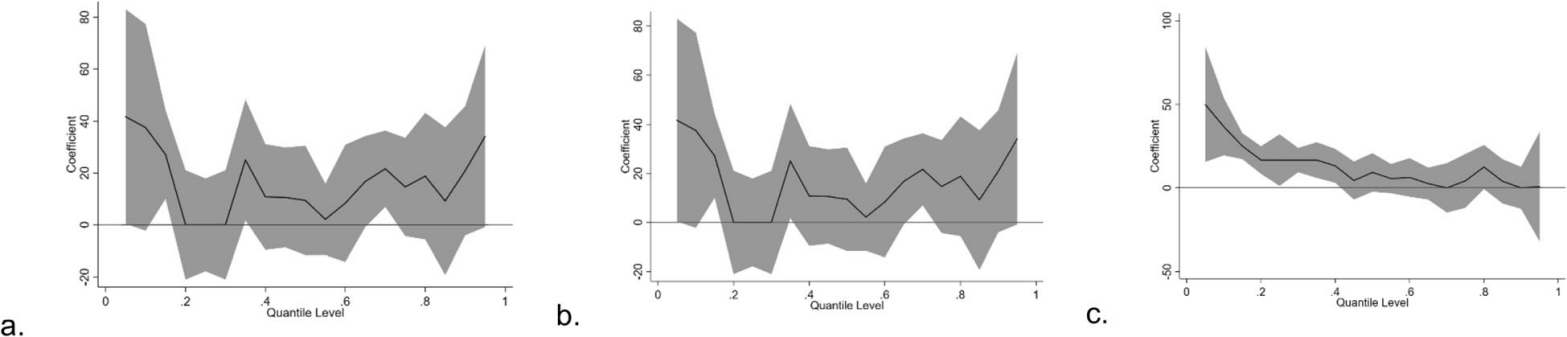
Graphical illustration of the effect of iron supplementation on birth weight in different percentiles before pregnancy and in the first trimester (**a**), in the second trimester (**b**) and in the third trimester (**c**)

Figure 2 shows the association between iron supplementation and the BW at different periods. The OLS found that iron supplementation had positive effects on the BW at different stages, with an average increase of 17.18 g (β = 17.18, t = 0.83, and *p* = 0.407; before pregnancy and the first trimester), 16.35 g (β = 16.35, t = 1.97, and *p* = 0.049; the second trimester), and 12.25 (β = 12.25, t = 2.08, and *p* = 0.037; the third trimester). The QR found that iron supplementation during pregnancy was associated with an increased newborn BW from very low to higher percentiles before pregnancy and in the first trimester (Fig. 2a, quantiles: 0 ~ 0.1, β = 133.33 ~ 50.00), in the second trimester (Fig. 2b, quantiles: 0.05, 0.15, 0.35, and 0.7, β = 41.67 ~ 21.60), and in the third trimester (Fig. 2c, quantiles: 0 ~ 0.4, β = 50.00 ~ 13.19). The detailed results are shown in table s1.

### Sensitivity analysis

**Fig. 3:**
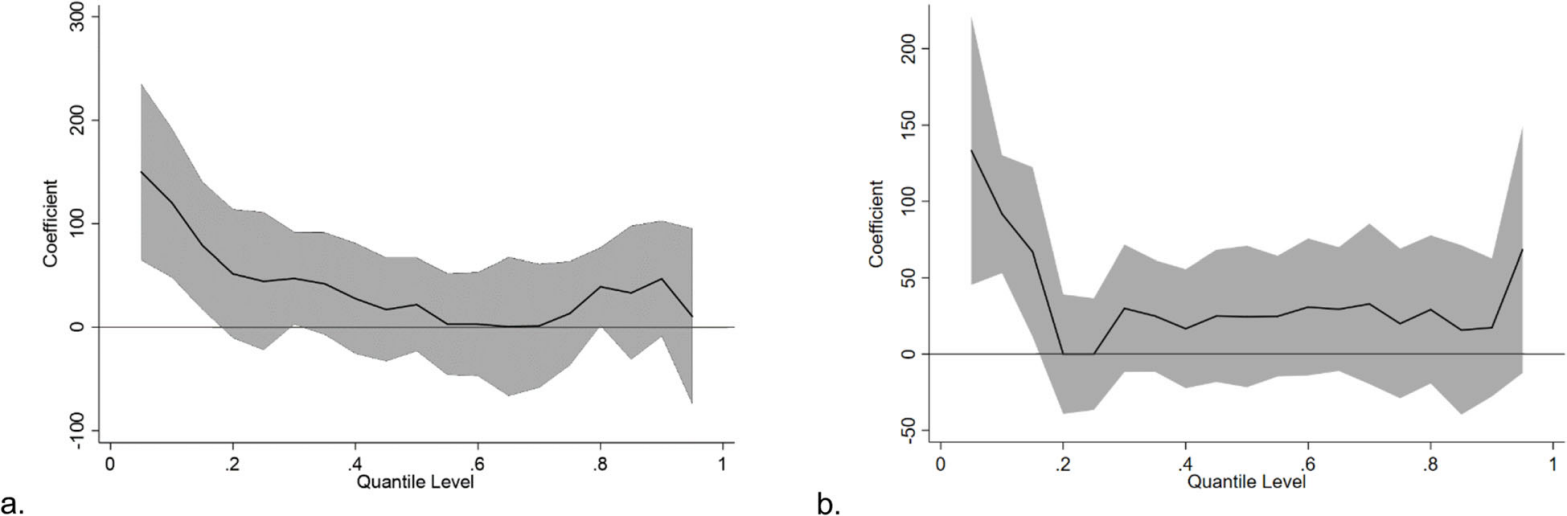
Graphical illustration of the effect of iron supplementation on birth weight in different percentiles for women suffered from anemia (**a**) and those not suffered from anemia (**b**)

To further analyze the effect of iron supplementation on the BW among different populations, stratified analysis was performed according to the anemia status. Among the women who suffered from anemia during pregnancy, the OLS method indicated that iron supplementation during pregnancy significantly increased the BW of newborns, with an average increase of 45.84 g (β = 45.84, t = 2.05, and *p* = 0.04), which revealed that iron supplementation was more effective among women who suffered from anemia. The QR showed that iron supplementation had positive effects on the BW from very low to higher percentiles (quantiles: 0 ~ 0.15, quantile: 0.30, quantile: 0.80), with β ranging from 150.00 to 39.29 (Fig. 3a). Among women without anemia, the multiple linear regression analysis showed an average increase of 38.18 g of the BW in newborns from the iron supplementation group (β = 38.18, t = 2.62, and *p* = 0.009). The QR showed that the effect of iron supplementation on the newborn BW was mainly concentrated in low percentiles (quantiles: 0 ~ 0.15), with β ranging from 133.33 to 28.32 (Fig. 3b). The effect was more obvious in the newborns with the lower BW than in those with the higher BW across the two groups. The detailed results are shown in table s2.

## Discussion

### Main findings

A low prevalence of women taking iron supplements (5.15%) was found in this study, which may be due to the lack of conventional iron supplementation guidelines for pregnant women in China [29]. We also found that the iron supplementation during pregnancy had a positive effect on the BW, with an average increase of 43.07 g (β = 43.07, t = 3.55, and *p* < 0.001). The QR confirmed this conclusion and further explored the effect at different BW percentiles. The QR showed that the effect of iron supplementation on BW was mainly concentrated in the low birth weight (quantiles: 0 ~ 0.40), with β ranging from 136.51 to 43.86 (*P* < 0.05). As the percentiles of BW increased, the BW gain of newborns with the iron supplementation gradually decreased compared with those without the iron supplementation. The OLS found that the association between iron supplementation before pregnancy and in the first trimester and the BW was not statistically significant; however, the QR showed that iron supplementation in this period had a positive effect on the low birth weight (quantiles: 0 ~ 0.1). The QR also found that the effect of iron supplementation during the second and third trimesters on the BW was mainly observed in the low birth weight group. The positive influence of iron supplementation on the BW is especially significant among women who suffered from anemia during pregnancy.

The QR could reveal the relationship between influencing factors and BW at different percentiles and more stably show the influence of the independent variables on both ends of the birth weight distribution. The QR provided more information and robust results than what had been previously shown.

### Comparison with other studies

Some studies regarding the association of iron supplementation with BW were conducted elsewhere. Batool A Haider et al. found that the iron intake significantly reduced the incidence of low birth weight (RR: 0.81, 95% CI: 0.71 ~ 0.93) through a review of a series of eligible RCTs and prospective cohort studies [30]. A comprehensive meta-analysis that included 60 randomized or quasi-randomized trials showed that the newborns whose mothers received iron were less likely to suffer from low birth weight (RR: 0.81; 95% CI: 0.68 ~ 0.97) and had increased mean birth weight by 30.81 g (95% CI: 5.94 ~ 55.68) [31]. Aamer Imdad et al. reported that routine daily iron supplementation significantly reduced the incidence of low birth weight by 20% (RR: 0.80, 95% CI: 0.71 ~ 0.90) and increased the mean birthweight by 42.18 g (95% CI: 9.27 ~ 75.09) [22]. The aforementioned studies are consistent with our study. There are few studies on iron supplementation in women with mild and moderate anemia or iron deficiency. A randomized clinical trial among rural Kenyan women indicated that iron supplementation increased the birth weight by 234 g (95% CI: 116 ~ 351 g) in women with iron deficiency [32], which is higher than the increase in our study (234 g vs 43.07 g). The etiologies of the LBW are complex and unclear and may vary in different settings. Several other factors that may influence the BW, such as infections, diet, poverty, and women’s status, may explain the differences reported in various studies [33].

### Strengths and limitations

The large sample size and representation were the main advantages of this investigation. The 28,209 newborns included in this study were all obtained by systematic sampling, accounting for approximately 9% of the newborns in the study area [34]. Considering the similar cultural diversities and lifestyle differences between Shaanxi Province and Northwest China, the results could be extended to Northwest China to some extent. In addition, the birth weight data obtained from the birth certificates were measured to the nearest 10 g, which is relatively accurate. Moreover, QR, a semiparametric robust regression technique, was used in our study to elucidate the association between iron supplementation and the BW at different percentiles, thus avoiding the OLS underestimating or overestimating the effect of iron supplementation during pregnancy on both ends of the BW range.

Although these findings were significant, some limitations in our data also must be considered. First, the study was retrospective, and all data were self-reported by the participants. Although previous studies have shown that events and nutritional supplementation during pregnancy could be well recalled even after a few years [35], the recall bias was inevitable. We took a series of measures to help women accurately recall to minimize the bias. On the one hand, before the formal investigation, we trained our interviewers rigorously on the uniform guidelines and performed a presurvey to test the survey tools. On the other hand, we collected information using standard questionnaires. Second, the lack of information on the frequencies and dosages of iron supplementation limited us to further perform quantitative analysis. Third, our study used the quantile regression, where the quantile selection was based on experience rather than the clinical derivation. Fourth, potential confounding factors, such as demographic characteristics and lifestyles, were adjusted in this study. However, some major factors previously reported to be associated with the BW, such as weight gain during pregnancy and pre-pregnancy BMI [33], were not included in this study. In particular, the lack of information about biomarkers of the body iron status limited our further exploration of the relationship between maternal iron status and the BW. We conducted a stratified analysis based on the anemia status to roughly explore this association. Nevertheless, as an exploratory study, the present research is the largest investigation in Shaanxi Province that reveals the impact of iron supplementation on the BW specific to this geographical region.

## Conclusions

Iron supplementation during pregnancy is associated with an increased newborn BW, and the effect was more obvious in the newborns with lower BW and the newborns whose mothers suffered from anemia during pregnancy. Fewer women took iron supplements during pregnancy in Shaanxi Province. Healthcare providers should provide iron supplementation guidelines for pregnant women, especially those with iron deficiency or anemia.

## Supplementary Information

**Additional file 1: Table S1.** QR and OLS results for accessing the association between iron supplementation and birth weight in different periods. **Table S2.** QR and OLS results for accessing the association between iron supplementation and birth weight depend on anemia status.

## Data Availability

The data used and/or analyzed during the current study are not publicly available. And it is available from the corresponding author on reasonable request.
