# Supplementary material for: Association between maternal iron supplementation and newborn birth weight: a quantile regression analysis
Source: Ital J Pediatr. 2021 Jun 5;47:133. doi: 10.1186/s13052-021-01084-7 (PMC8180103; doi:10.1186/s13052-021-01084-7)
Supplement: Supplementary file 1 — Additional file 1: Table S1. QR and OLS results for accessing the association between iron supplementation and birth weight in different periods. Table S2. QR and OLS results for accessing the association between iron supplementation and birth weight depend on anemia status. [file 13052_2021_1084_MOESM1_ESM.docx]

**Title page**

**Association between maternal iron supplementation and newborn birth weight: a quantile regression analysis**

**Guoshuai Shi ^1^, Zhuo Zhang ^2^, Lu Ma ^3^, Binyan Zhang ^1^, Shaonong Dang ^1*^ and Hong Yan ^1,4,*^**

^1^Department of Epidemiology and Biostatistics, School of Public health, Xi’an Jiaotong University Health Science Center, Xi’an, Shaanxi, 710061, China.

^2^Institute of Chinese Medical Sciences & State Key Laboratory of Quality Research in Chinese Medicine, University of Macau, Macau, China.

^3^ Xi'an Shiyou University, Xi’an, Shaanxi, 710065, China.

^4^Nutrition and Food Safety Engineering Research Center of Shaanxi, Xi’an, Shaanxi, 710061, China.

^*^ Correspondence: xjtu_yhpaper@126.com (H.Y.); tjdshn@xjtu.edu.cn (S.D.)

**Table S1** QR and OLS results for accessing the association between iron supplementation and birth weight in different periods

| **Before pregnancy and the first trimester** | | | | | **The second trimester** | | | | **The third trimester** | | | |
| --- | --- | --- | --- | --- | --- | --- | --- | --- | --- | --- | --- | --- |
| **Quantile** | **β** | **95% CI** | **t** | **P values** | **β** | **95% CI** | **t** | **P values** | **β** | **95% CI** | **t** | **P values** |
| 0.05 | 133.33 | 87.97~178.69 | 5.76 | 0.00 | 41.67 | 0.31~83.02 | 1.97 | 0.05 | 50.00 | 15.57~84.43 | 2.85 | 0.00 |
| 0.10 | 50.00 | 15.73~84.27 | 2.86 | 0.00 | 37.50 | -2.31~77.31 | 1.85 | 0.07 | 36.67 | 19.48~53.85 | 4.18 | 0.00 |
| 0.15 | 27.78 | -7.13~62.69 | 1.56 | 0.12 | 27.08 | 9.97~44.2 | 3.10 | 0.00 | 25.00 | 17.2~32.8 | 6.28 | 0.00 |
| 0.20 | 0.00 | -35.23~35.23 | 0.00 | 1.00 | 0.00 | -21.04~21.04 | 0.00 | 1.00 | 16.67 | 8.4~24.93 | 3.95 | 0.00 |
| 0.25 | 0.00 | -44.29~44.29 | 0.00 | 1.00 | 0.00 | -17.85~17.85 | 0.00 | 1.00 | 16.67 | 1.22~32.12 | 2.11 | 0.04 |
| 0.30 | 0.00 | -38.53~38.53 | 0.00 | 1.00 | 0.00 | -21.03~21.03 | 0.00 | 1.00 | 16.67 | 9.36~23.97 | 4.47 | 0.00 |
| 0.35 | 0.00 | -40.23~40.23 | 0.00 | 1.00 | 25.00 | 1.64~48.36 | 2.10 | 0.04 | 16.67 | 5.98~27.35 | 3.06 | 0.00 |
| 0.40 | -15.00 | -73.43~43.43 | -0.50 | 0.62 | 10.71 | -9.66~31.09 | 1.03 | 0.30 | 13.19 | 3.02~23.37 | 2.54 | 0.01 |
| 0.45 | -10.53 | -79.84~58.79 | -0.30 | 0.77 | 10.52 | -8.74~29.78 | 1.07 | 0.28 | 4.41 | -7~15.82 | 0.76 | 0.45 |
| 0.50 | 1.69 | -53.45~56.84 | 0.06 | 0.95 | 9.38 | -11.74~30.49 | 0.87 | 0.38 | 9.26 | -2.31~20.83 | 1.57 | 0.12 |
| 0.55 | -12.50 | -60.96~35.96 | -0.51 | 0.61 | 2.08 | -11.67~15.84 | 0.30 | 0.77 | 5.56 | -3.21~14.32 | 1.24 | 0.21 |
| 0.60 | 0.00 | -52.61~52.61 | 0.00 | 1.00 | 8.33 | -14.23~30.9 | 0.72 | 0.47 | 6.25 | -5.32~17.82 | 1.06 | 0.29 |
| 0.65 | 0.00 | -72.32~72.32 | 0.00 | 1.00 | 16.67 | -0.91~34.24 | 1.86 | 0.06 | 2.56 | -7.01~12.14 | 0.52 | 0.60 |
| 0.70 | 0.00 | -62.68~62.68 | 0.00 | 1.00 | 21.60 | 6.88~36.31 | 2.88 | 0.00 | 0.00 | -14.81~14.81 | 0.00 | 1.00 |
| 0.75 | 6.25 | -70.58~83.08 | 0.16 | 0.87 | 14.58 | -4.39~33.55 | 1.51 | 0.13 | 4.17 | -11.96~20.3 | 0.51 | 0.61 |
| 0.80 | 30.00 | -56.34~116.34 | 0.68 | 0.50 | 18.75 | -5.68~43.18 | 1.50 | 0.13 | 12.50 | -0.74~25.74 | 1.85 | 0.06 |
| 0.85 | 25.00 | -49.74~99.74 | 0.66 | 0.51 | 9.15 | -19.3~37.59 | 0.63 | 0.53 | 3.94 | -9.33~17.21 | 0.58 | 0.56 |
| 0.90 | 0.00 | -62.9~62.9 | 0.00 | 1.00 | 20.83 | -4.09~45.76 | 1.64 | 0.10 | 0.00 | -12.59~12.59 | 0.00 | 1.00 |
| 0.95 | 29.00 | -72.41~130.41 | 0.56 | 0.58 | 34.09 | -0.9~69.08 | 1.91 | 0.06 | 0.72 | -32.17~33.62 | 0.04 | 0.97 |
| OLS | 17.18 | -23.45~57.8 | 0.83 | 0.41 | 16.35 | 0.1~32.6 | 1.97 | 0.05 | 12.25 | 0.72~23.79 | 2.08 | 0.04 |

**Table S2** QR and OLS results for accessing the association between iron supplementation and birth weight depend on anemia status

| **Anemia** | | | | | **No- anemia** | | | | |
| --- | --- | --- | --- | --- | --- | --- | --- | --- | --- |
| **Quantile** | **β** | **95% CI** | **t** | **P values** | **Quantile** | **β** | **95% CI** | **t** | **P values** |
| 0.05 | 150.00 | 64.81~235.19 | 3.45 | 0.00 | 0.05 | 133.33 | 45.45~221.22 | 2.97 | 0.003 |
| 0.10 | 120.35 | 48.34~192.36 | 3.28 | 0.00 | 0.10 | 91.78 | 53.2~130.36 | 4.66 | 0.000 |
| 0.15 | 78.89 | 17.24~140.53 | 2.51 | 0.01 | 0.15 | 66.88 | 11.36~122.4 | 2.36 | 0.018 |
| 0.20 | 51.73 | -10.42~113.88 | 1.63 | 0.10 | 0.20 | 0.00 | -39.23~39.23 | 0.00 | 1.000 |
| 0.25 | 44.53 | -21.98~111.05 | 1.31 | 0.19 | 0.25 | 0.00 | -36.62~36.62 | 0.00 | 1.000 |
| 0.30 | 47.35 | 2.84~91.87 | 2.09 | 0.04 | 0.30 | 30.00 | -11.68~71.68 | 1.41 | 0.158 |
| 0.35 | 42.11 | -7.2~91.42 | 1.67 | 0.09 | 0.35 | 25.00 | -11.59~61.59 | 1.34 | 0.181 |
| 0.40 | 27.78 | -25.62~81.17 | 1.02 | 0.31 | 0.40 | 16.67 | -22.35~55.69 | 0.84 | 0.402 |
| 0.45 | 17.15 | -32.84~67.14 | 0.67 | 0.50 | 0.45 | 25.00 | -18.28~68.28 | 1.13 | 0.258 |
| 0.50 | 22.02 | -23.09~67.13 | 0.96 | 0.34 | 0.50 | 24.59 | -21.71~70.89 | 1.04 | 0.298 |
| 0.55 | 3.13 | -46.04~52.29 | 0.12 | 0.90 | 0.55 | 24.81 | -14.66~64.29 | 1.23 | 0.218 |
| 0.60 | 3.08 | -47.13~53.29 | 0.12 | 0.90 | 0.60 | 30.86 | -13.95~75.67 | 1.35 | 0.177 |
| 0.65 | 0.60 | -66.43~67.64 | 0.02 | 0.99 | 0.65 | 29.43 | -11.04~69.9 | 1.43 | 0.154 |
| 0.70 | 1.47 | -58.4~61.34 | 0.05 | 0.96 | 0.70 | 32.92 | -19.63~85.47 | 1.23 | 0.220 |
| 0.75 | 13.49 | -36.79~63.77 | 0.53 | 0.60 | 0.75 | 20.00 | -28.96~68.96 | 0.80 | 0.423 |
| 0.80 | 39.29 | 1.68~76.89 | 2.05 | 0.04 | 0.80 | 29.21 | -19.26~77.69 | 1.18 | 0.238 |
| 0.85 | 33.33 | -31.24~97.91 | 1.01 | 0.31 | 0.85 | 15.77 | -39.69~71.22 | 0.56 | 0.577 |
| 0.90 | 47.01 | -8.76~102.78 | 1.65 | 0.10 | 0.90 | 17.39 | -27.72~62.5 | 0.76 | 0.450 |
| 0.95 | 10.53 | -74.28~95.34 | 0.24 | 0.81 | 0.95 | 68.32 | -12.36~149 | 1.66 | 0.097 |
| OLS | 45.84 | 2.07~89.61 | 2.05 | 0.04 | OLS | 38.18 | 9.65~66.71 | 2.62 | 0.009 |
